# Supplementary material for: Functional Role of AGAP2/PIKE-A in Fcγ Receptor-Mediated Phagocytosis
Source: Cells. 2022 Dec 24;12(1):72. doi: 10.3390/cells12010072 (PMC9818964; doi:10.3390/cells12010072)
Supplement: Supplementary file 1 [file cells-12-00072-s001.zip › cells-2075474-supplementary.pdf]

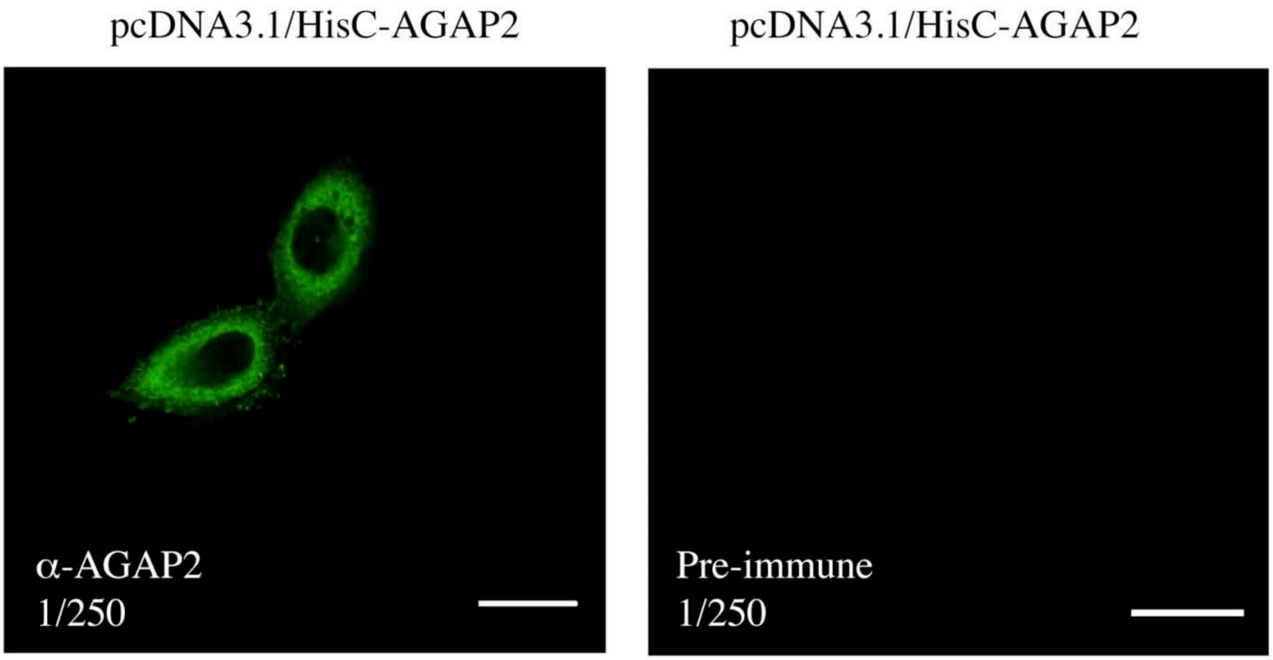

Supplemental Figure S1. Localization of pcDNA3.1/HisC-AGAP2 in CHO-IIA cells. CHO-IIA cells transiently transfected with pcDNA3.1/HisC-AGAP2 were labeled with anti-AGAP2 antibody 1/250 (left panel) or with the pre-immune serum at the same concentration (right panel). Cells were examined under confocal microscopy at the same settings and a single confocal cross section of cells is represented. Bars, 20 μm.
